# A Manufacturing-Oriented Intelligent Vision System Based on Deep Neural Network for Object Recognition and 6D Pose Estimation

**DOI:** 10.3389/fnbot.2020.616775

**Published:** 2021-01-07

**Authors:** Guoyuan Liang, Fan Chen, Yu Liang, Yachun Feng, Can Wang, Xinyu Wu

**Affiliations:** ^1^Center for Intelligent and Biomimetic Systems, Shenzhen Institutes of Advanced Technology, Chinese Academy of Sciences, Shenzhen, China; ^2^Guangdong Provincial Key Lab of Robotics and Intelligent System, Shenzhen Institutes of Advanced Technology, Chinese Academy of Sciences, Shenzhen, China; ^3^Shenzhen Key Laboratory of Human-Machine Intelligence-Synergy Systems, Shenzhen Institutes of Advanced Technology, Chinese Academy of Sciences, Shenzhen, China; ^4^Guangdong-Hong Kong-Macao Joint Laboratory of Human-Machine Intelligence-Synergy Systems (No.2019B121205007), Shenzhen Institutes of Advanced Technology, Chinese Academy of Sciences, Shenzhen, China

**Keywords:** deep neural network, object recognition, 6D pose estimation, intelligent manufacturing, semantic segmentation

## Abstract

Nowadays, intelligent robots are widely applied in the manufacturing industry, in various working places or assembly lines. In most manufacturing tasks, determining the category and pose of parts is important, yet challenging, due to complex environments. This paper presents a new two-stage intelligent vision system based on a deep neural network with RGB-D image inputs for object recognition and 6D pose estimation. A dense-connected network fusing multi-scale features is first built to segment the objects from the background. The 2D pixels and 3D points in cropped object regions are then fed into a pose estimation network to make object pose predictions based on fusion of color and geometry features. By introducing the channel and position attention modules, the pose estimation network presents an effective feature extraction method, by stressing important features whilst suppressing unnecessary ones. Comparative experiments with several state-of-the-art networks conducted on two well-known benchmark datasets, YCB-Video and LineMOD, verified the effectiveness and superior performance of the proposed method. Moreover, we built a vision-guided robotic grasping system based on the proposed method using a Kinova Jaco2 manipulator with an RGB-D camera installed. Grasping experiments proved that the robot system can effectively implement common operations such as picking up and moving objects, thereby demonstrating its potential to be applied in all kinds of real-time manufacturing applications.

## 1. Introduction

The assembly line is one of the greatest inventions in the manufacturing industry. With the rapid development of artificial intelligence and robotics, more intelligent robotics have been deployed in traditional assembly lines, replacing human workers. These robots are normally equipped with intelligent vision systems which not only detect parts in the working spaces but also estimate their poses before taking further actions such as grasping, rotating, moving, fitting, etc. Generally, object recognition and 6D pose estimation from images are the base of almost all kinds of robotic applications, such as robot manipulation (Tremblay et al., 2018), robot-human interaction (Svenstrup et al., 2009), and virtual reality (Yang et al., 2018).

Many approaches have been reported in the past decade. However, the problem remains challenging, especially in cluttered scenes due to the chaos in backgrounds, heavy occlusions between objects, and changing lighting conditions. Most classical methods work with RGB inputs (color images) (Kehl et al., 2017; Rad and Lepetit, 2017), and some of them use RGB-D inputs (color and depth images) (Wagner et al., 2008). In general, the basic idea of these methods is to estimate the object pose by establishing the correspondence of 2D image features between different viewpoints or constructing mapping from 3D models to 2D images. Difficulties often occur when dealing with low-textured objects and unstable lighting conditions. With the advent of affordable depth sensors, RGB-D data-based methods (Xiang et al., 2017; Qi et al., 2018; Xu et al., 2018) have become more popular and has recently undergone significant progress. Compared with the RGB image, the depth image contains abundant geometric information, such as shape, structure, surface, curvature, etc. Additionally, the depth channel is more stable than RGB channels, usually free from perturbance caused by texture and the changing of light, which makes these approaches more reliable and robust than RGB-only methods. However, due to involvement of a large amount of 3D data, it is still a big challenge to achieve accurate pose estimation in real-time.

With the appearance of powerful computing hardware, deep learning has garnered wide attention in recent years. (Tekin et al., 2018) proposed a single-shot deep CNN that takes the 2D image as the input, directly detects the 2D projections of the 3D bounding box vertices and estimates 6D poses by a PnP algorithm (Lepetit et al., 2009). Based on SSD architecture (Liu et al., 2016), SSD-6D (Kehl et al., 2017) can realize object detection and 6D pose estimation simultaneously, though it does not work well with the small objects and occlusions. Most recently, a series of data-driven methods using RGB-D inputs such as PoseCNN (Xiang et al., 2017), MCN (Li et al., 2018a), and DenseFusion (Wang et al., 2019) were presented and has made significant progress in the field of visual recognition. In addition, some methods, such as PointFusion (Xu et al., 2018) and Frustrum PointNet (Qi et al., 2018), focus on how to extract better features from color and depth images. Compared with methods based on handcraft-features, the deep neural network demonstrates a powerful ability for automatic feature extraction, a flexible structure, and an amazing capacity of resisting disturbance.

**Figure 1:**
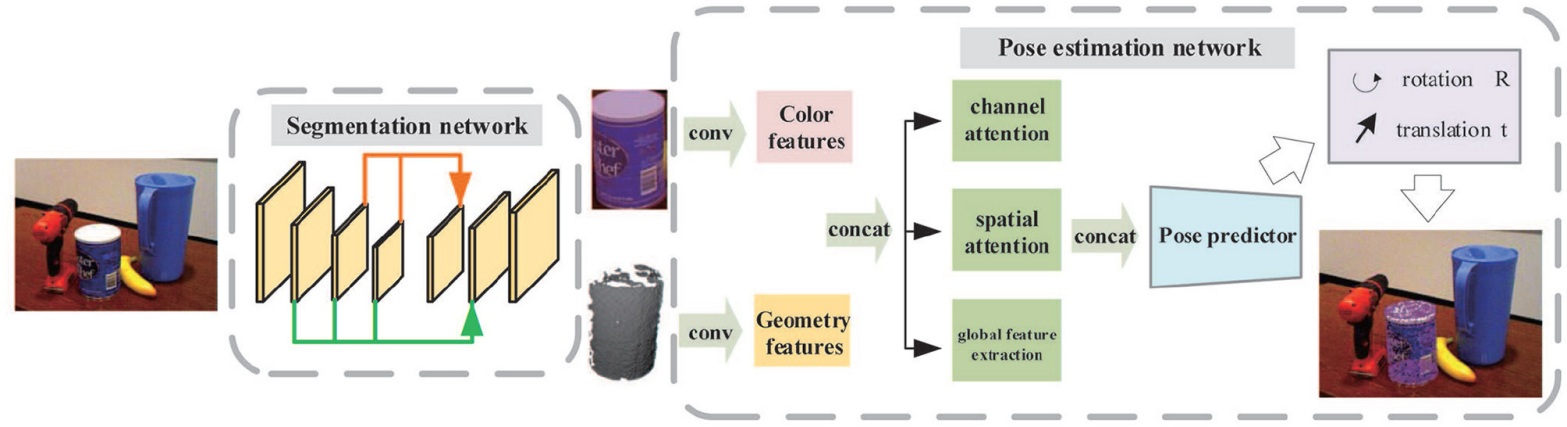
The overall framework of the two-stage network for object recognition and pose estimation.

In this paper, we propose a new two-stage deep network to segment objects from the cluttered scene and to estimate their 6D poses. The overall framework is shown in Figure 1. First, by applying a dense-connected way to aggregate features of different scales, an improved segmentation network inspired by U-Net (Ronneberger et al., 2015) is built. After determining the segmentation mask, the objects are cropped from the scene in both color and depth images. The cropped object images are then fed into a 6D pose prediction network which makes use of two backbone networks to extract color and geometry feature embeddings. Both are then fused together and pass through the channel attention module, position attention module, and global feature extraction module to acquire more effective feature representations. Finally, an iterative network is adopted to refine outputs of the pose predictor.

In summary, the main contributions of our approach are stated as follows:

- A new segmentation network is proposed using a densely connected method to aggregate features of different scales and to provide abundant semantic information for pixel-by-pixel classification.
- The channel attention module and position attention module are introduced into the pose estimation network and effectively improve system performance.
- A vision-guided robotic grasping system is built to validate the feasibility of the proposed algorithm being applied to manufacturing applications like grasping, packaging, and assembling etc.

The remainder of this paper is organized as follows: section 2 reviews related work. Section 3 describes the details of the proposed method. The analysis of experimental results and performance evaluation are presented in section 4. Section 5 concludes the paper.

## 2. Related Work

Our research mainly involves two topics: object recognition and pose estimation. Semantic segmentation is the most popular way to realize object recognition by predicting which object each pixel in the image belongs to. For semantic segmentation, Convolutional Neural Network (CNN) has proven to be the most successful method so far. For object pose estimation, prior works can be classified into three categories: feature-based methods, learning-based methods, and CNN-based methods. Theoretically, CNN is a specific framework in the machine learning family. A boom in research on deep learning has led to a dramatic number of CNN-based methods being reported in recent years. We therefore placed the CNN-based methods into a separate category.

### 2.1. Semantic Segmentation

Fully Convolutional Network (FCN) (Long et al., 2015) is the first semantic segmentation network. (Ronneberger et al., 2015) added skip connections to the network and created an excellent network known as U-Net. Many subsequent networks (Drozdzal et al., 2016) adopted this U-shape structure to develop their own networks. In order to increase the area of the receptive field without extra computing costs, Atrous Convolutions are proposed in the Deeplab (Chen et al., 2017a). In PSPNet (Zhao et al., 2017), the Pyramid Pooling Module (PPM) is proposed to aggregate contextual information from different scales to improve the network's ability to acquire global information.

### 2.2. Object Pose Estimation

#### 2.2.1. Feature-Based Method

The common idea for the feature-based methods is recovering 6D poses based on 2D-3D correspondences (Tulsiani and Malik, 2015) by matching the local features extracted in the 2D image with those in the 3D model. However, this kind of approach usually requires sufficient texture in objects. Some improved versions of this algorithm (Wohlhart and Lepetit, 2015) are proposed to deal with textureless objects.

#### 2.2.2. Learning-Based Method

Machine learning has been widely applied to address classical computer vision problems since 2000. Support Vector Machine (SVM) is proposed in Gu and Ren (2010) for object pose estimation. Hinterstoisser presented a model-based method (Hinterstoisser et al., 2012) to classify objects and 3D poses as well. In Lee et al. (2016), an adaptive Bayesian framework is designed to implement object detection and pose estimation in industrial environments. A decision forest is trained in Brachmann et al. (2014) to classify each pixel in the RGB-D image and determine the postures of objects.

#### 2.2.3. CNN-Based Method

Most recently, with the rapid development of deep learning, CNN has become the mainstream approach in most pose estimation tasks. Zeng proposed a multi-stage feature learning network in Zeng et al. (2016) for object detection and pose estimation with RGB-D inputs. Literatures (Rad and Lepetit, 2017; Tekin et al., 2018) predicted the 2D projections of 3D bounding box of a 3D target by regression before computing poses by PnP algorithm. Kehl localized the 2D bounding box of an object and searched for the best pose from a pool of candidate 6D poses associated with the bounding box (Kehl et al., 2017). Nigam improved the accuracy of pose estimation in Nigam et al. (2018) through a novel network architecture which combined global features for target segmentation with local features for coordinate regression. Li adapted a CNN-based framework by adding a channel for 3D feature extraction (Li et al., 2018a). PoseCNN (Xiang et al., 2017) is a multi-stage, multi-branch deep network that takes the 6D object poses estimation as a regression problem. With RGB images as inputs, the network can directly estimate the object poses in the whole scene. DenseFusion (Wang et al., 2019) is a general framework for estimating the 6D poses of known objects with RGB-D image inputs. Two networks are utilized to extract color and geometric features, followed by a novel dense network fusing them. In addition, an end-to-end iterative refinement network is also applied further improving the pose estimations. In sum, DenseFusion is one of the best networks so far, perfectly balancing accuracy and efficiency, thus making it appropriate for many real-time manufacturing applications.

## 3. Theory and Method

As illustrated in Figure 1, the proposed system consists of two stages: object segmentation and object pose estimation. The final objective is to recover object pose parameters from 2D-3D correspondences between the color and depth image. Therefore, an appropriate camera model should be determined before calculation.

### 3.1. Pinhole Camera Model

**Figure 2:**
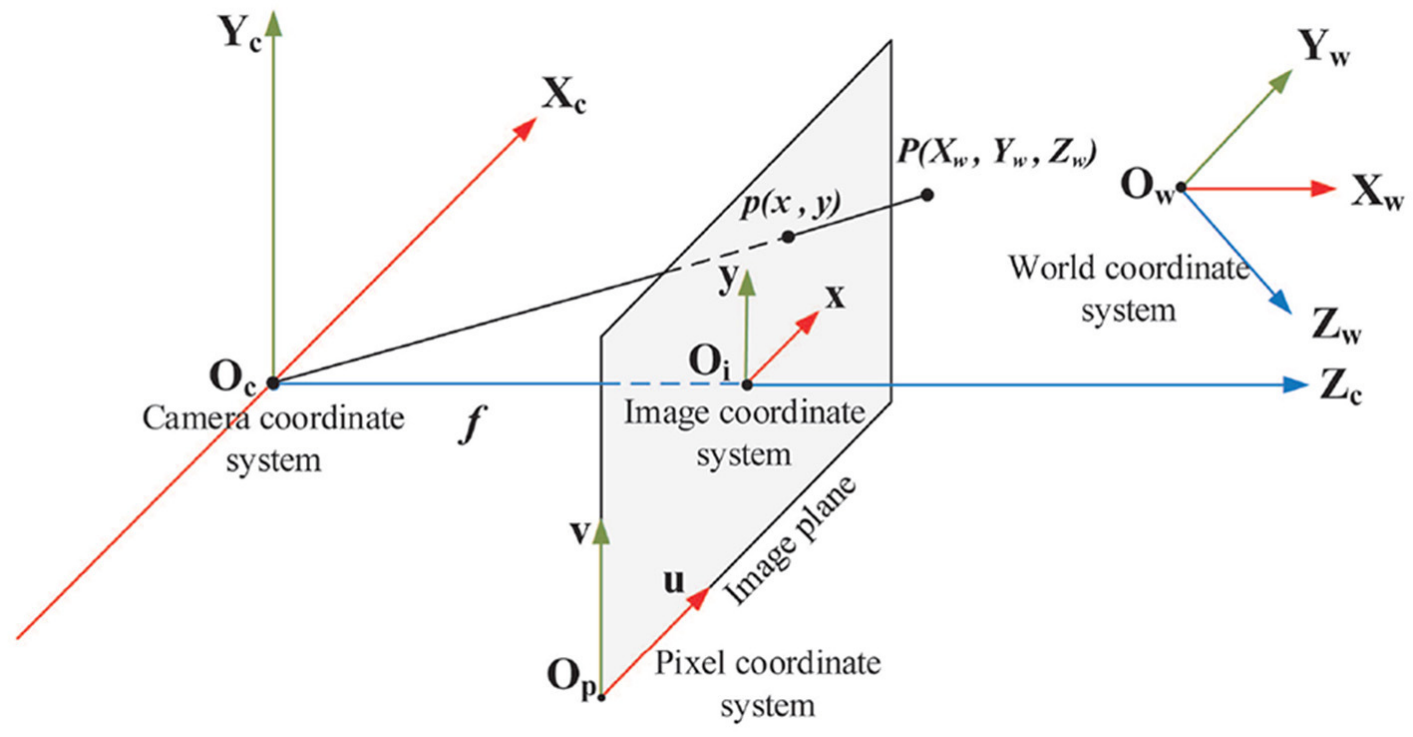
Four coordinate systems in the pinhole camera model: world coordinate system, camera coordinate system, image coordinate system and pixel coordinate system. *P*(*X*_*w*_, *Y*_*w*_, *Z*_*w*_) is a 3D point and *p*(*x, y*) is the projection of *P*(*X*_*w*_, *Y*_*w*_, *Z*_*w*_) in the image plane. *f* is the focal length that is the length between origin of camera coordinate system and origin of image coordinate system.

Figure 2 shows the concept of the pinhole camera model with four coordinate systems.

- **World coordinate system (*X*_*w*_, *Y*_*w*_, *Z*_*w*_)** is the absolute coordinate system of 3D world, also named as the object coordinate system in our application.
- **Camera coordinate system (*X*_*c*_, *Y*_*c*_, *Z*_*c*_)** is a 3D coordinate system with camera optical center as the origin, cameras optical axis as *Z*_*c*_-axis, *X*_*c*_-axis and *Y*_*c*_-axis parallel to *x*-axis and *y*-axis of image coordinate system respectively.
- **Image coordinate system (*x, y*)** is a 2D coordinate system located in the image plane with the intersection of *Z*_*c*_-axis and image sensor as origin, *x*-axis and *y*-axis parallel to the horizontal and vertical edges of image plane respectively. The 2D coordinates denote pixel position in physical units, such as millimeters.
- **Pixel coordinate system (*u, v*)** is a 2D coordinate system with the bottom-left corner of image plane as origin, *u*-axis and *v*-axis parallel to *x*-axis and *y*-axis of image coordinate system respectively. The digital image is represented by an *M* × *N* array of pixels. The 2D coordinates denote the pixel position in the image array.

By geometric analysis, the transformation between pixel coordinate system and world coordinate system can be expressed in homogeneous form by

(1) $$\begin{array}{l} Z_{c}\left(\begin{array}{c} u\\ v\\ 1 \end{array}\right)=\left(\begin{array}{ccc} \frac{1}{dx} & 0 & u_{0}\\ 0 & \frac{1}{dy} & v_{0}\\ 0 & 0 & 1 \end{array}\right)\left(\begin{array}{cccc} f & 0 & 0 & 0\\ 0 & f & 0 & 0\\ 0 & 0 & 1 & 0 \end{array}\right)\left(\begin{array}{cc} R & t\\ 0^{T} & 1 \end{array}\right)\left(\begin{array}{c} X_{w}\\ Y_{w}\\ Z_{w}\\ 1 \end{array}\right)\\ \;=\left(\begin{array}{cccc} a_{x} & 0 & u_{0} & 0\\ 0 & a_{y} & v_{0} & 0\\ 0 & 0 & 1 & 0 \end{array}\right)\left(\begin{array}{cc} R & t\\ 0^{T} & 1 \end{array}\right)\left(\begin{array}{c} X_{w}\\ Y_{w}\\ Z_{w}\\ 1 \end{array}\right)\\ \;=M_{1}M_{2}\left(\begin{array}{c} X_{w}\\ Y_{w}\\ z_{w}\\ 1 \end{array}\right)=M\left(\begin{array}{c} X_{w}\\ Y_{w}\\ z_{w}\\ 1 \end{array}\right) \end{array}$$

where (*u*_0_, *v*_0_) are the pixel coordinates of the origin of the image coordinate system in the pixel coordinate system; *dx* and *dy* are the physical dimensions of each pixel in the *x*-axis and *y*-axis of the image plane respectively; *f* is the focal length; *R* is a 3 × 3 orthogonal rotation matrix, and *t* is a 3D translation vector indicating the transformation from the world coordinate system to the camera coordinate system. It can be seen that the parameters of matrix *M*_1_ are determined by the internal structure of the camera sensor. Thus, *M*_1_ is called an intrinsic parameter matrix of the camera. The rotation matrix *R* and translation vector *t*, however, are determined by the position and orientation of the camera coordinate system relative to the world coordinate system. *M*_2_ is called the extrinsic parameter matrix of the camera which is determined by three rotation and three translation parameters. Parameters in *M*_1_ can be calculated through camera calibration. Solving six pose parameters in *M*_2_ is actually called the 6D pose estimation.

### 3.2. Semantic Segmentation

**Figure 3:**
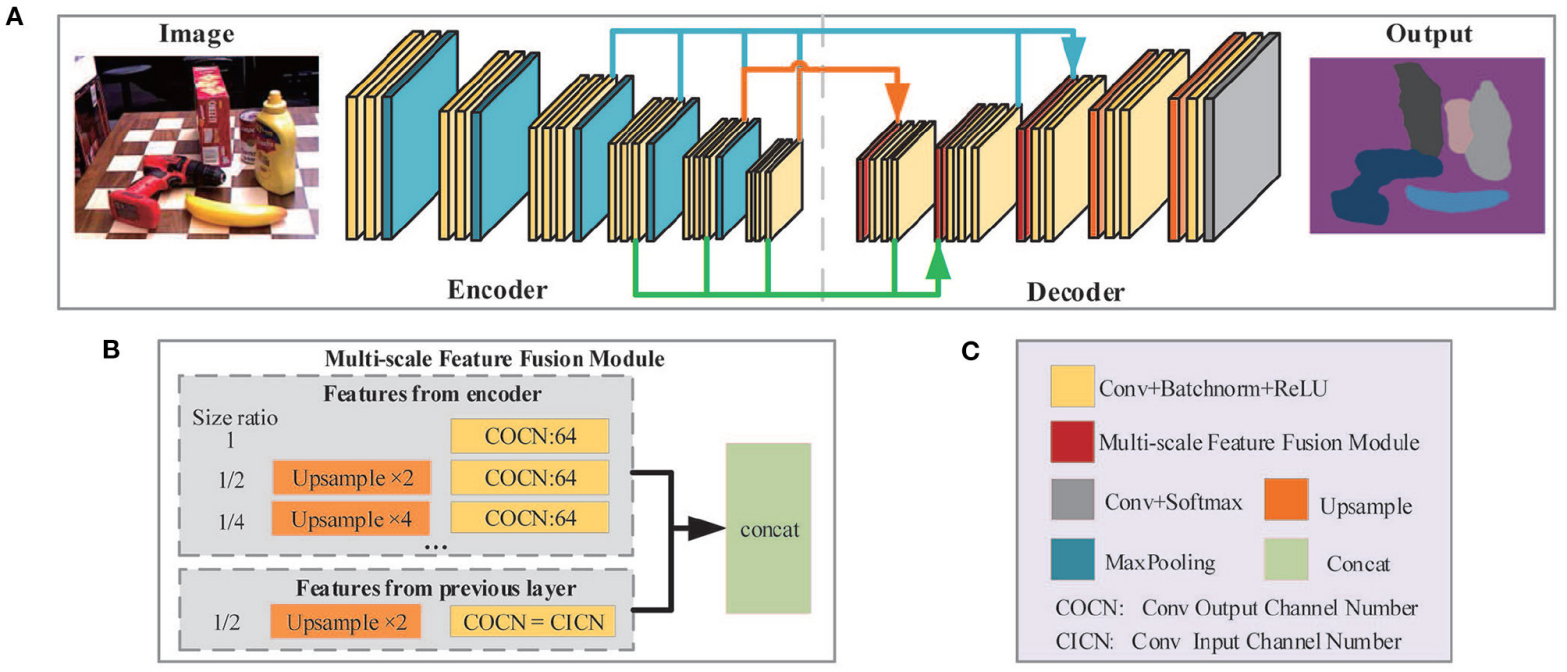
The framework of semantic segmentation network. **(A)** Network architecture. VGG16 is utilized to extract features from the image while MFFM is applied to aggregate feature maps from different layers. **(B)** The structure of MFFM. **(C)** Legends for **(A,B)**.

The architecture of the semantic segmentation network is illustrated in Figure 3. The entire network is composed of two parts: the encoder network (Figure 3A, left) and the decoder network (Figure 3A, right). The encoder network is designed to extract features of different scales, which consists of five MaxPooling layers and 16 convolutional layers. Same parameter settings such as the convolutional layer in VGG16 (Simonyan and Zisserman, 2015) are applied in the first 13 convolutional layers. In the decoder network, the Multi-scale Feature Fusion Module (MFFM) is implemented to fuse multi-scale features and output pixel-by-pixel classifications through the final convolutional and softmax layer. The decoder network consists of three MFFMs, two upsampling layers as well as several convolution layers.

In convolutional neural networks, feature maps of different sizes not only have diverse receptive fields but also contain complementary information generally. Therefore, fusing features of different scales is an important technique to improve network performance. Researchers have proposed different solutions for multi-scale feature extraction and fusion. Deeplabv3 (Chen et al., 2017b) utilized Atrous Spatial Pyramid Pooling (ASPP) to fuse global content information with multi-scale features. In Zhao et al. (2017), the global AvgPooling operation is applied to generate different scale outputs from feature maps and to extract high-level semantic multi-scale features. However, these methods parallelly convolute and pool the same feature layer at different scales to acquire multi-scale features. In essence, it is not a real fusion of different layer features since these features are all extended from the same layer. Theoretically, a lower-layer feature contains more geometric detail and less semantic information. Conversely, higher-layer feature maps discard some geometric detail and preserve more semantic information. Thus, a new MFFM is designed to effectively integrate lower-layer and higher-layer features through a densely connected way, thereby enhancing the network's capability of understanding the images.

As shown in Figure 3B, each MFFM layer in the decoder takes the feature inputs from two sources: (1) the layers in the encoder with the same or a lower resolution of the current MFFM layer; (2) the previous layer in the decoder. First, all feature inputs will be upsampled to the resolution of the current MFFM layer, if necessary. Then each of them will pass through an individual convolution layer before finally being aggregated together and output. For inputs from encoder layers, the output channel number is set to 64 to reduce computational complexity. For inputs from the previous layer, the output channel number remains unchanged in order to preserve information coming from the previous layer as much as possible. Thus, different MFFM layers may have various numbers of input layers, as depicted in Figure 3A by arrows in different colors.

In the training stage, the lost function of the segmentation network is defined as cross-entropy and is expressed by

(2) $$\begin{array}{r} Loss=-\overset{H}{\underset{i=1}{\sum }}\overset{W}{\underset{j=1}{\sum }}y_{i,j}^{{}^{\prime }}\log y_{i,j}, \end{array}$$

where *y*_*i,j*_ ∈ {1, 2, …, *C*} is the true label of each pixel and $y_{i,j}^{{}^{\prime }}$ is the prediction.

In sum, the proposed semantic segmentation network provides more effective pixel-by-pixel classification by, in a densely connected way, fusing multi-scale features. Despite the small increase in computational cost, more accurate pixel classifications are helpful in determining the correct correspondences between 2D pixels and 3D points, which is crucial for the second stage of 6D pose estimation. Furthermore, the segmentation network can also extract the correct object contour, showing its ability in dealing with occlusions under complex scenes.

### 3.3. 6D Object Pose Estimation

**Figure 4:**
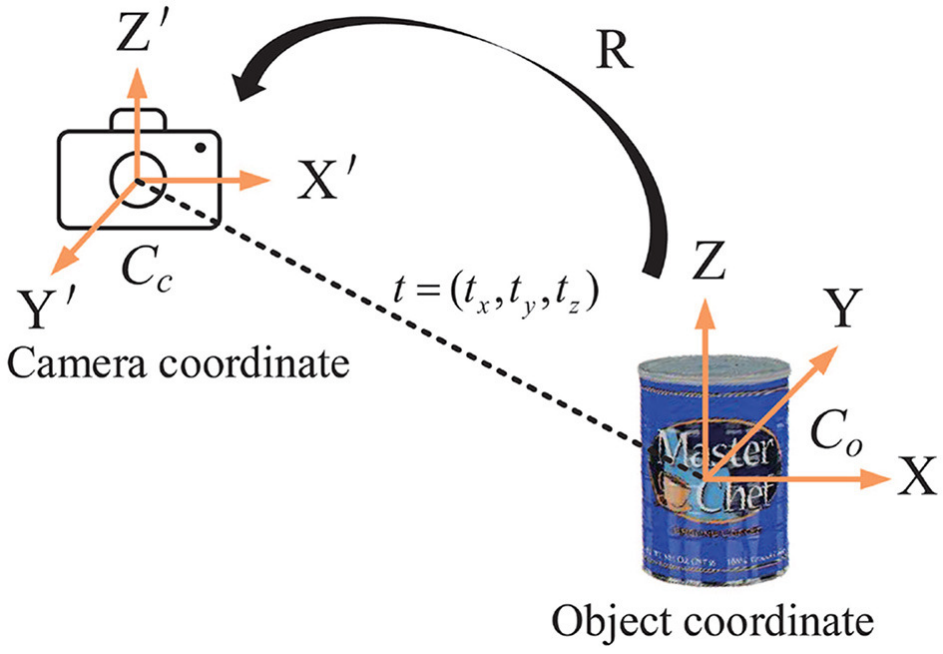
Object 6D pose estimation. The pose transformation from the object coordinate system to the camera coordinate system is determined by the 3D rotation matrix *R* and the 3D translation vector *t*.

As mentioned in section 3.1, object pose estimation determines the transform between the object coordinate system and the camera coordinate system. A 3D translation vector *t* and a 3D rotation matrix *R* are included in the transformation matrix. So, there are six independent pose parameters to be calculated. Figure 4 depicts the concept.

**Figure 5:**
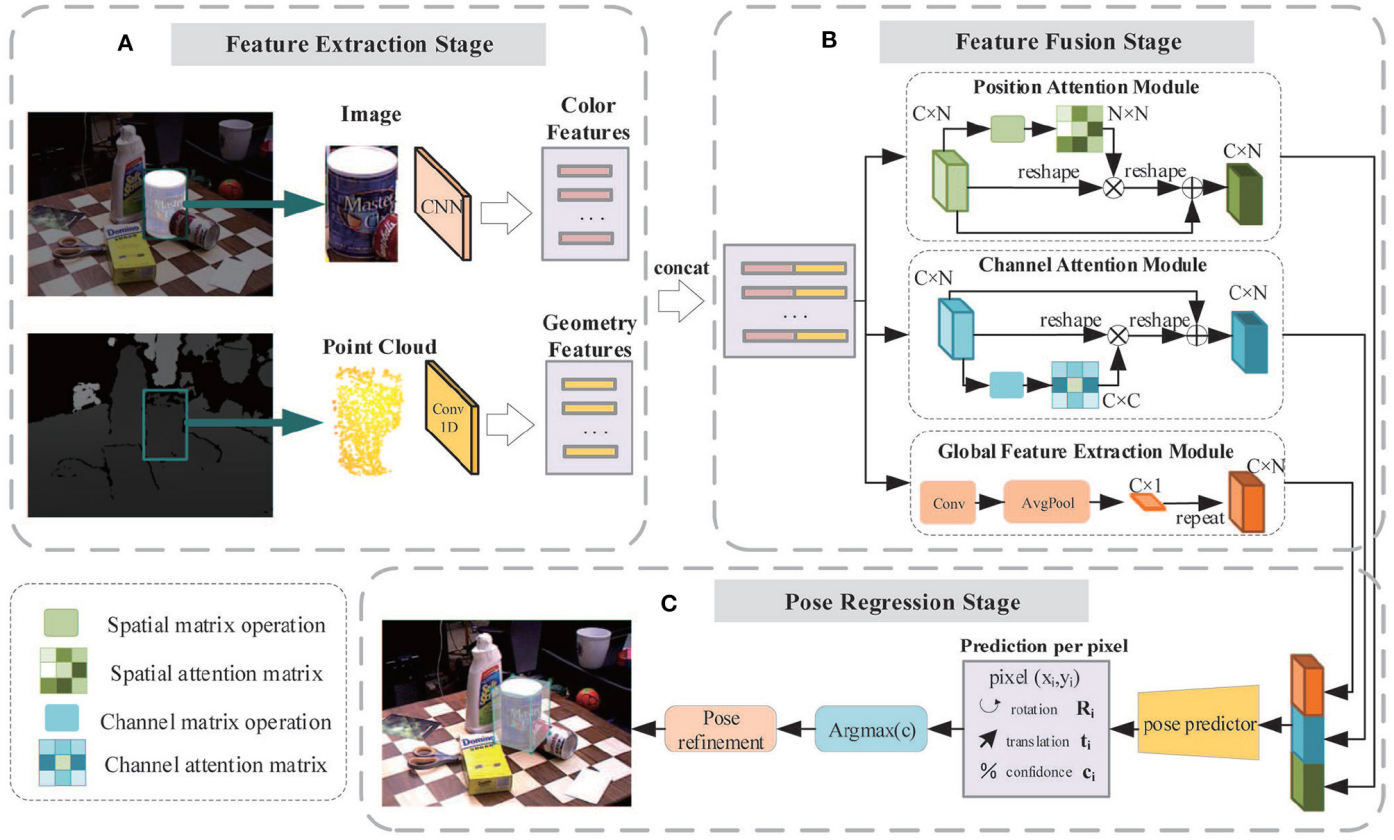
The framework of the three-stage 6D pose estimation network. **(A) Feature extraction stage:** The color feature embedding is extracted by a full convolution network and the geometric feature embedding is extracted by a PointNet-based network; **(B) Feature fusion stage:** Two feature embeddings are fused together and then pass through the channel attention module, position attention module, and global feature extraction module, respectively, to generate three types of features. All of them are fused and fed to the pose predictor. **(C) Pose regression stage:** The pose predictor consisting of several 1D convolutions is utilized to regress the 6D pose parameters and confidence scores.

The architecture of our 6D pose estimation network is illustrated in Figure 5. The entire network is composed of three stages: feature extraction stage (Figure 5A), feature fusion stage (Figure 5B), and pose regress stage (Figure 5C).

The color and geometric information of objects are acquired through the RGB image and depth image. Though they have similar storage formats, the physical meaning and distribution space are quite different. Two CNNs are applied to extract color and geometric features, respectively, as shown in Figure 5A.

Common neural networks generally treat all the features equally. However, some features are better at describing characteristics of objects and should receive more attention. To stress important features whilst suppressing unnecessary ones, we implemented three modules including the Position Attention Module (PAM), Channel Attention Module (CAM), and the Global Feature Extraction Module (GFEM). In the feature fusion stage, color features and geometric features are concatenated and fed into these modules, enabling the network to adaptively capture the local features and the global feature dependencies, providing better features for the pose predictor.

**Figure 6:**
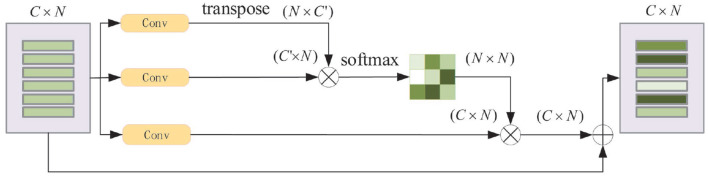
Schematic diagram of position attention module. *N* is number of the features and *C* is the feature dimension.

**Position Attention Module:** For a specified input feature, it is updated by weighting all the features according to their similarity to this feature. Thus, more similar features will have a bigger impact on the input feature. Figure 6 displays the process.

The original input feature matrix *V*_*i*_ ($V_{i}\in R^{C\times N}$, where C is the feature dimension and N is the number of the features), passes through two convolutional layers separately and get two new feature matrices *V*_1_, *V*_2_ ($V_{1},V_{2}\in R^{C^{\prime }\times N}$. The dimension of *V*_1_, *V*_2_' changes from *C* to *C*′ after passing through, in this paper, we set $C^{\prime }=\frac{C}{8}$). After transposing *V*_1_ multipling *V*_2_, and followed by a softmax operation, the feature similarity matrix *M* (*M* ∈ *R*^*N* × *N*^) is obtained. In the meantime, *V*_*i*_ passes through the third convolution layer to obtain *V*_3_ ($V_{3}\in R^{C\times N}$), it is then multiplied by *M* to aggregate global features. Finally, the output features *V*_*o*_ are calculated as *V*_*o*_ = *V*_3_ × *M* + *V*_*i*_.

**Figure 7:**
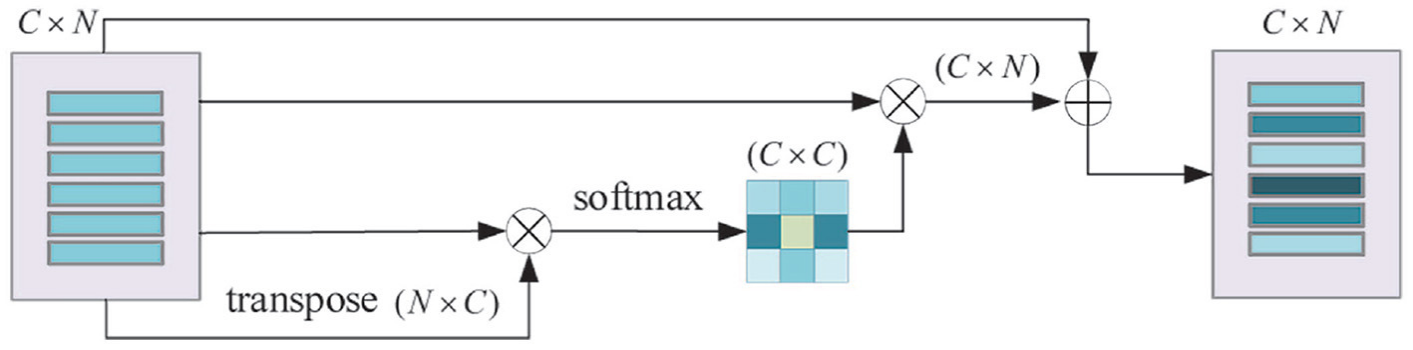
Schematic diagram of channel attention module. *N* is the number of features and *C* is the feature dimension.

**Channel Attention Module:** For any two channel maps, the self-attention mechanism is used to capture the channel dependencies. The weighted sum of all channel maps is calculated to update each channel map. The process of the channel attention module is shown in Figure 7.

**Global Feature Extraction Module:** The global feature of the objects is quite important for the pose estimation task. Here we use a convolutional layer to adjust the features and apply an AvgPooling layer to acquire the global features.

The output features from the PAM, CAM, and GFEM are concatenated and fed into a pose predictor. The pose predictor consists of several 1D convolution layers for regressing 6D pose parameters and confidence scores. In addition, the same iterative refinement network as DenseFusion (Wang et al., 2019) is also utilized for further improvement in accuracy of pose estimation.

Due to the lack of a unique shape or texture associated with poses for the objects with symmetric structures, we use two types of loss functions in the training stage. For symmetric objects, the loss for one point in the estimated model is defined as the distance between this point and its closest point after pose transformation. The total loss of an object with *n* points is computed as follows:

(3) $$L_{sym}=\frac{1}{n}\sum_{i=1}^{n}\underset{i<k<n}{\min}\left\|T_{est}p_{i}-T_{gt}p_{k}\right\|.$$

where *T* = [*R*|*t*], *R* and *t* are rotation matrix and translation vector, respectively. Meanwhile, *p*_*i*_ represents the homogeneous form of *i*^*th*^ point. *T*_*est*_ is the current predicted result and *T*_*gt*_ is the ground truth. For the object with an asymmetric structure, each pose is associated with a unique texture or shape. So, the loss function of the asymmetric object is defined as the average distance of each point after real pose transformation and the predicted pose transformation, as described in Eq. (4). In the training stage, we switch the loss function according to the labels given with the dataset.

(4) $$\begin{array}{r} L_{asy}=\frac{1}{n}\overset{n}{\underset{i=1}{\sum }}||T_{est}p_{i}-T_{gt}p_{i}||. \end{array}$$

## 4. Experimental Results and Analysis

### 4.1. Experiment Setup

#### 4.1.1. The Platform of Hardware and Software

The proposed network in this paper is built by Pytorch (Paszke et al., 2019). All the experiments are conducted on a PC equipped with an Intel(R) Core(TM) i7-6850k CPU at 3.6GHz and a NVIDIA GeForce GTX1080Ti graphic card.

#### 4.1.2. Training Setup

For the segmentation network, the initial learning rate is set to 0.0001, batch size is 4. The Stochastic Gradient Descent (SGD) optimizer is used in the training. The learning rate decreased by 0.8 times for every 5,000 training steps.

For the pose estimation network, the initial learning rate is set to 0.0001, batch size is 8. The Adam optimizer (Kingma and Ba, 2015) is used in the training. When the test error drops to a certain threshold of 0.14, the learning rate starts to decline 0.3 times. When the test error drops to 0.12, the iterative refinement network starts to kick in the training process.

### 4.2. Datasets and Metrics

#### 4.2.1. Datasets

Two well-known benchmark datasets, the YCB-video (Xiang et al., 2017) and the LineMOD (Hinterstoisser et al., 2011), were employed in our experiments.

**The YCB-Video dataset** is built from Xiang et al. (2017) for object pose estimation. The dataset is composed of 92 RGB-D video sequences taken in different indoor scenes, and 21 objects with different shapes and textures. Here we follow the same training and testing settings as the PoseCNN (Xiang et al., 2017) where 80 video sequences are used for training, and 2,949 key frames extracted from the remaining 12 videos are used to evaluate the trained model. In addition, 80,000 synthetically rendered images provided by the dataset are also utilized to augment the training set.

**The LineMOD dataset** (Hinterstoisser et al., 2011) is another benchmark dataset for object pose estimation, which contains 15 video sequences of low-texture objects. There are approximately 1,100 images in each video sequence. For a fair comparison, we choose the same 13 video sequences, and the same training and testing set as some state-of-the-art methods (Kehl et al., 2017; Rad and Lepetit, 2017; Xiang et al., 2017; Li et al., 2018b; Sundermeyer et al., 2018; Xu et al., 2018). Besides, no synthetic data are generated from the LineMOD dataset and used for training.

#### 4.2.2. Metrics

**Metrics for semantic segmentation**. Two common metrics, Mean Pixel Accuracy (mPA) and Mean Intersection over Union (mIoU) (Badrinarayanan et al., 2017; Garcia-Garcia et al., 2017) are used to evaluate the segmentation results. The mPA is defined as the mean of pixel accuracy for all classes, where the pixel accuracy is computed by the ratio of correct pixels on a per-class basis. The mIoU is defined as the mean of IoUs for all classes in the dataset, where IoU for each class is computed as the ratio of the intersection and the union of label pixels and predicted pixels.

**Metrics for pose estimation**. We adopted three metrics to evaluate system performance on the YCB-Video dataset, namely, ADD-S (Xiang et al., 2017) (the average closest point distance), ADD (the average distance) (Hinterstoisser et al., 2012), and AUC (Xiang et al., 2017) (the area under the accuracy-threshold curve of ADD/ADD-S). Given the ground truth pose *P* (*P* = [*R*|*T*]) and estimated pose $\tilde{P}$ ($\tilde{P}\;=\;\left[\tilde{R}|\tilde{T}\right]$), we can calculate the distance between the point in the 3D model transformed by $\tilde{P}$ and the nearest point to it in the 3D model transformed by *P*. The average distance of all points is called ADD-S. ADD has a similar definition as ADD-S, the difference is that the distance is calculated from corresponding point pairs in the 3D model transformed by $\tilde{P}$ and *P*. In our experiments, the accuracy, defined as the percentage of testing samples with ADD/ADD-S values less than a certain threshold, is used to evaluate the performance of all methods. Here, the threshold is empirically set to 2 cm. The accuracy values for all possible thresholds are calculated and an accuracy-threshold curve of ADD/ADD-S is generated. AUC is then defined as the area under this curve within the threshold region [0, *T*_*m*_]. To have a consistent measurement, the maximum threshold *T*_*m*_ is set to 0.1 m. For AUC and accuracy, a larger value indicates better accuracy of pose estimation.

For LineMOD dataset, ADD (Hinterstoisser et al., 2012) is used as the metric following prior works (Kehl et al., 2017; Rad and Lepetit, 2017; Xiang et al., 2017; Li et al., 2018b; Sundermeyer et al., 2018; Xu et al., 2018). Instead of a fixed value, we set the distance threshold as 0.1 multiplied by the diameter of bounding sphere of the 3D model.

### 4.3. Experiments on YCB-Video Dataset

**Semantic segmentation:** Table 1 shows the comparative experiment results of pose estimation on the YCB-Video Dataset where bold numbers represent the best results for each metric. It is observed that our network is much better than the U-Net in both mPA and mIoU. Moreover, our mPA score is a little bit smaller than Deeplabv3 whilst our mIoU score outperforms Deeplabv3 by 2.78.

**Table 1:** Quantitative evaluation of semantic segmentation on YCB-Vedio dataset.

	**mPA(%)**	**mIoU(%)**
U-Net	58.02	48.01
Deeplabv3	**84.85**	76.28
Ours	84.28	**79.06**

*Bold values represent the best results for all metrics*.

**Figure 8:**
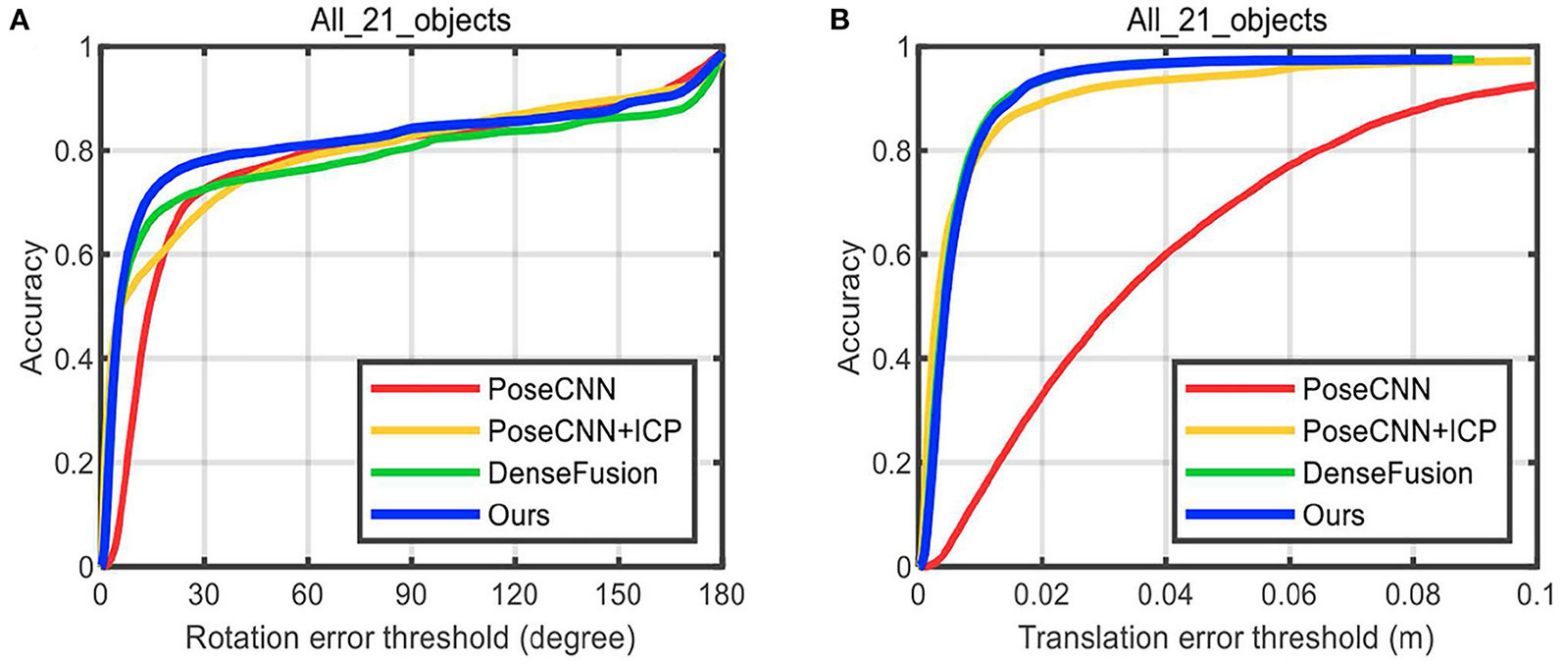
The accuracy-threshold curves of pose parameter error. **(A)** The accuracy-threshold curve of rotation angle error, **(B)** The accuracy-threshold curve of translation error.

**6D Pose estimation:** Tables 2, 3 show the comparative experiment results of pose estimation on 21 objects in the YCB-Video Dataset using our method and some state-of-the-art methods, where the bold numbers represent the best results. In Table 2, AUC and accuracy of ADD-S (<2 cm) are calculated for all objects. As seen from the table, our mean accuracy score is the second-best and the mean AUC score is the best. The overall performance is very close to DenseFusion which is the benchmark method thus far. In Table 3, AUC and accuracy of ADD (<2 cm) are calculated. Apparently both mean accuracy score and mean AUC score of the proposed network outperform all the other methods. Essentially, ADD is a better and stricter metric than ADD-S because it computes the distances between matched point pairs, which usually requires matches on both shape and texture. A better ADD accuracy score is more convincible in evaluating the performance of our method. Figure 8A displays the accuracy-threshold curve of the rotation error and Figure 8B displays the accuracy-threshold curve of the translation error. The rotation error is the angle formed by the predicted and true rotation axes. The translation error is the 2-Norm of the difference between the predicted and true translation vectors. Accuracy is therefore defined as the percentage of testing samples with fewer translation/rotation errors than a threshold. For each threshold, the corresponding accuracy is calculated to build the accuracy-threshold curve. Figures 8A,B exhibit the superior accuracy of our method in both rotation and translation predictions. Moreover, a steeper curve is observed near zero degrees in the accuracy-threshold curve of the rotation error, showing that the proposed method can achieve higher accuracy at a small rotation error threshold, which indicates the smaller pose estimation errors.

**Table 2:** Quantitative evaluation of 6D pose estimation (ADD-S) on YCB-Video Dataset.

	**PoseCNN**		**PoseCNN+ICP**		**PointFusion**		**DenseFusion**		**Ours**	
**Object**	**AUC**	**<2 cm**	**AUC**	**<2 cm**	**AUC**	**<2 cm**	**AUC**	**<2 cm**	**AUC**	**<2 cm**
002 master chef can	83.9	71.5	95.8	**100.0**	90.9	99.8	**96.4**	**100.0**	96.1	**100.0**
003 cracker box	76.9	56.6	92.7	91.6	80.5	62.6	95.5	99.5	**96.1**	**99.9**
004 sugar box	84.2	71.2	**98.2**	**100**	90.4	95.4	97.5	**100.0**	97.2	**100.0**
005 tomato soup can	81.0	74.4	94.5	**96.9**	91.9	**96.9**	**94.6**	**96.9**	94.2	**96.9**
006 mustard bottle	90.4	95.8	**98.6**	**100.0**	88.5	84.0	97.2	**100.0**	97.2	**100.0**
007 tuna fish can	88.0	84.8	**97.1**	99.7	93.8	99.8	96.6	**100.0**	96.4	**100.0**
008 pudding box	79.1	58.4	**97.9**	**100.0**	87.5	96.7	96.5	**100.0**	96.6	**100.0**
009 gelatin box	87.2	89.7	**98.8**	**100.0**	95.0	**100.0**	98.1	**100.0**	97.5	**100.0**
010 potted meat can	78.5	68.0	**92.7**	**93.6**	86.4	88.5	91.3	93.1	90.9	93.0
011 banana	86.0	84.2	**97.1**	99.7	84.7	70.5	96.6	**100.0**	95.6	99.7
019 pitcher base	77.0	38.8	**97.8**	100.0	85.5	79.8	97.1	**100.0**	97.0	**100.0**
021 bleach cleanser	71.6	39.7	**96.9**	99.4	81.0	65.0	95.8	**100.0**	96.0	**100.0**
024 bowl	69.6	14.0	81.0	54.9	75.7	24.1	**88.2**	**98.8**	87.4	83.5
025 mug	78.2	58.5	94.9	99.8	94.2	99.8	**97.1**	**100.0**	97.1	99.5
035 power drill	72.7	53.1	**98.2**	**99.6**	71.5	22.8	96.0	98.7	96.1	99.5
036 wood block	64.3	8.3	87.6	80.2	68.1	18.2	**89.7**	**94.6**	86.9	79.3
037 scissors	56.9	18.2	91.7	95.6	76.7	35.9	**95.2**	**100.0**	94.0	**100.0**
040 large marker	71.7	46.1	97.2	99.7	87.9	80.4	**97.5**	**100.0**	97.0	**100.0**
051 large clamp	50.2	31.7	75.2	74.9	65.9	50.0	72.9	**79.2**	**73.3**	78.5
052 extra-large clamp	44.1	17.6	64.4	48.8	60.4	20.1	69.8	**76.3**	**73.6**	72.4
061 foam brick	88.0	87.5	**97.2**	**100.0**	91.8	**100.0**	92.5	**100.0**	95.3	**100.0**
**Mean**	75.8	58.2	93.0	93.2	83.9	74.1	93.1	**96.8**	**93.2**	96.0

*Bold values represent the best results for all metrics*.

**Table 3:** Quantitative evaluation of 6D pose estimation (ADD) on YCB-Video Dataset.

	**PoseCNN**		**PoseCNN+ICP**		**DenseFusion**		**Ours**	

**Object**	**AUC**	<**2 cm**	**AUC**	<**2 cm**	**AUC**	<**2 cm**	**AUC**	<**2 cm**
002 master chef can	50.2	8.25	68.1	51.1	73.2	**72.8**	**73.4**	**72.8**
003 cracker box	53.1	13.0	83.4	73.3	94.2	98.2	**94.4**	**99.1**
004 sugar box	68.4	41.1	**97.2**	99.5	96.5	**100.0**	95.6	99.9
005 tomato soup can	66.2	42.9	81.8	76.6	85.4	82.9	**89.5**	**89.8**
006 mustard bottle	81.0	62.8	**98.0**	98.6	94.8	96.1	95.5	**100.0**
007 tuna fish can	70.7	47.3	**83.9**	**72.1**	81.9	62.8	79.8	60.5
008 pudding box	62.7	25.7	**96.6**	**100.0**	93.2	98.6	94.5	**100.0**
009 gelatin box	75.2	32.7	**98.1**	**100.0**	96.7	**100.0**	96.0	**100.0**
010 potted meat can	59.5	30.4	83.5	77.9	**83.6**	79.8	**82.0**	80.0
011 banana	72.3	31.4	**91.9**	88.1	83.5	**88.7**	75.6	79.2
019 pitcher base	53.3	12.1	**96.9**	97.7	**96.9**	99.8	95.9	**100.0**
021 bleach cleanser	50.3	11.4	**92.5**	**92.7**	90.1	90.4	90.7	90.6
024 bowl	3.33	0.0	**14.4**	**0.25**	5.85	0.00	7.59	0.0
025 mug	58.5	17.5	81.1	55.2	88.9	89.5	**92.0**	**92.6**
035 power drill	55.3	25.7	**97.7**	**99.2**	92.8	96.3	93.8	**99.2**
036 wood block	26.6	0.83	**70.8**	**64.9**	30.8	0.0	24.5	0.0
037 scissors	35.8	2.2	78.4	49.2	77.9	50.3	**87.8**	**85.1**
040 large marker	58.3	14.8	85.3	87.2	**93.0**	**100.0**	92.5	99.9
051 large clamp	24.6	3.7	**52.1**	36.4	26.4	0.0	40.5	**38.1**
052 extra-large clamp	16.1	2.9	26.5	8.2	24.6	16.6	**46.6**	**40.0**
061 foam brick	40.2	0.0	48.1	0.0	**59.1**	0.0	44.1	0.0
**MEAN**	53.7	23.3	79.2	71.3	78.0	73.7	**79.7**	**78.0**

*Bold values represent the best results for all metrics*.

**Figure 9:**
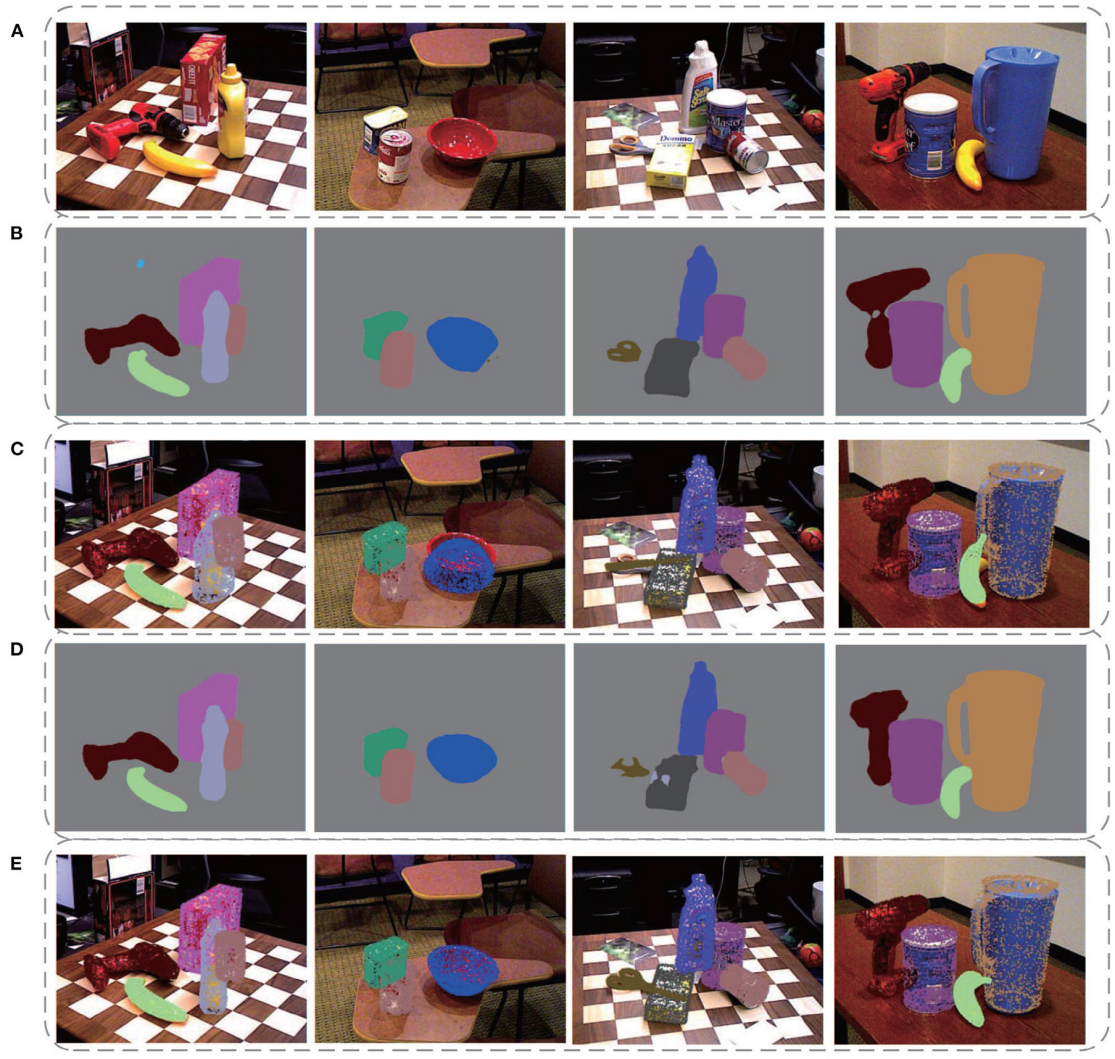
Some qualitative experimental results on the YCB-Video dataset. **(A)** The original images in the dataset, **(B)** Segmentation results of DenseFusion, **(C)** Pose estimation results of DenseFusion, **(D)** Segmentation results of our method, **(E)** Pose estimation results of our method.

Figure 9 displays some qualitative results on the YCB-Video dataset. Figure 9A is the original images in the dataset. Figures 9B,D are segmentation results of DenseFusion and our method. Different colors stand for different object categories here. After acquiring the segmentation mask, the pixel-level area of each object in the image is extracted. If the effective pixel number in the depth map of an object is less than a certain threshold, it is identified as an invalid object without estimating its poses. For each valid object, the point cloud is transformed with the predicted pose parameters. Its projection in the 2D image is then superimposed over the object region, as shown in Figures 9C,E. As illustrated in the second column from the left, the prediction for the bowl by DenseFusion is far away from its real orientation. Our method, however, provides a more correct prediction showing its advantage in dealing with symmetric objects. For some poor-textured objects, such as the banana in the first and fourth column, obvious errors are spotted for DenseFusion with no visually perceptible errors for our method.

**Time efficiency**. Table 4 shows the time efficiency comparison of our network with PoseCNN and DenseFusion. The time cost of all computation components including segmentation, pose estimation, and iterative refinement are calculated, respectively, for a more intuitive comparison except for PoseCNN, as it is not a pipeline structure network. For the total running time, our method is five times faster than PoseCNN. Compared with DenseFusion, our method is slightly slower in segmentation, while being slightly faster in pose estimation. Although the total time consumption is slightly lower than DenseFusion, it meets the requirements of real-time applications at a processing rate of 18 frames per second with about five objects in each frame. Considering the better accuracy of pose estimation, our method is overall proved to be the best among these state-of-the-art methods. What is more, a lightweight network will be applied for feature extraction in the future, which is expected to improve the time efficiency tremendously.

**Table 4:** Time efficiency of three methods (sec.).

**PoseCNN**	**DenseFusion**				**Ours**			
**Seg+PE **(**ALL**)****	**Seg**	**PE**	**Refine**	**ALL**	**Seg**	**PE**	**Refine**	**ALL**
0.283	0.035	0.010	0.002	0.047	0.045	0.009	0.002	0.056

*Seg, segmentation; PE, pose estimation; Refine, iterative refinement*.

### 4.4. Experiments on LineMOD Dataset

**Figure 10:**
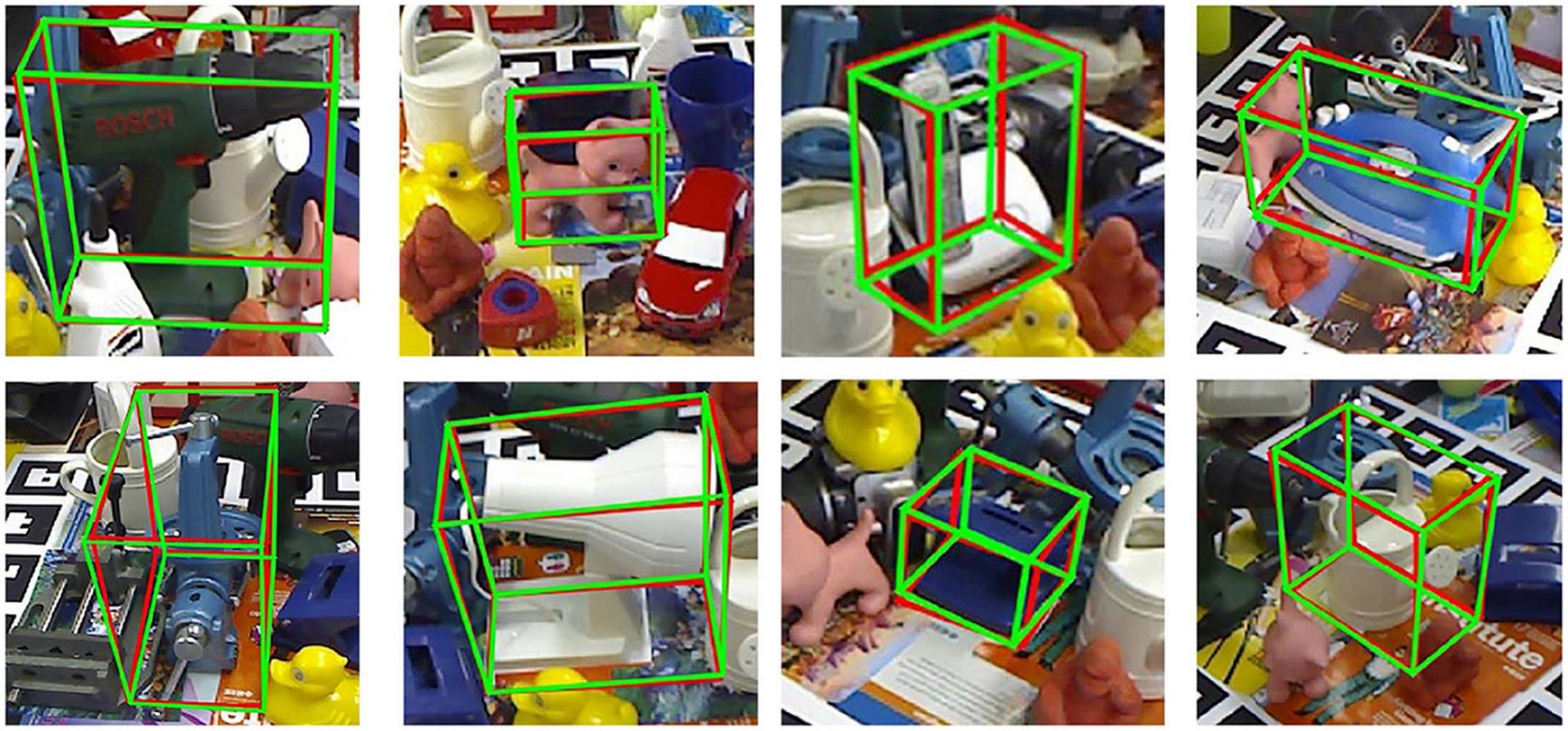
Pose estimation results of our method for some images with cluttered background in the LineMOD dataset. The red box and the green box are 2D projections of the 3D bounding box of objects which, transformed by true pose parameters and predicted ones, respectively.

Table 5 shows the comparison of our method with some other methods [BB8(Rad and Lepetit, 2017), PoseCNN+DeepIM (Xiang et al., 2017; Li et al., 2018b), Implicit (Sundermeyer et al., 2018)+ICP, SSD-6D (Kehl et al., 2017)+ICP, PointFusion (Xu et al., 2018), DenseFusion (Wang et al., 2019)] on the LineMOD Dataset with the accuracy of ADD (<2cm) adopted as metric. For the mean accuracy, our method outperforms DenseFusion by 2.6, Figure 10 visualizes the pose estimation results of our method on LineMOD Dataset. As expected, only small errors are perceived in these images even if under cluttered environments.

**Table 5:** Quantitative evaluation of 6D pose estimation (ADD) on LineMOD Dataset.

	**RGB**		**RGB-D**				
	**BB8**	**PoseCNN**	**Implicit**	**SSD-6D**	**Point Fusion**	**Dense Fusion**	**Ours**
**object**		**+DeepIM**	**+ICP**	**+ICP**			
ape	40.4	77.0	20.6	65	70.4	**92.3**	91.8
benchvise	91.8	97.5	64.3	80	80.7	93.2	**96.9**
camera	55.7	93.5	63.2	78	60.8	94.4	**98.3**
can	64.1	96.5	76.1	86	61.1	93.1	**96.9**
cat	62.6	82.1	72.0	70	79.1	96.5	**97.0**
driller	74.4	95.0	41.6	73	47.3	87.0	**94.7**
duck	44.3	77.7	32.4	66	63.0	92.3	**95.3**
eggbox	57.8	97.1	98.6	**100**	99.9	99.8	**100.0**
glue	41.2	99.4	96.4	**100**	99.3	100.0	**100.0**
holepuncher	67.2	52.8	49.9	49	71.8	92.1	**96.2**
iron	84.7	**98.3**	63.1	78	83.2	97.0	97.8
lamp	76.5	**97.5**	91.7	73	62.3	95.3	**97.5**
phone	54.0	87.7	71.0	79	78.8	92.8	**97.5**
MEAN	62.7	88.6	64.7	79	73.7	94.3	**96.9**

*Bold values represent the best results for all metrics*.

### 4.5. Vision-guided Robotic Grasping System

**Figure 11:**
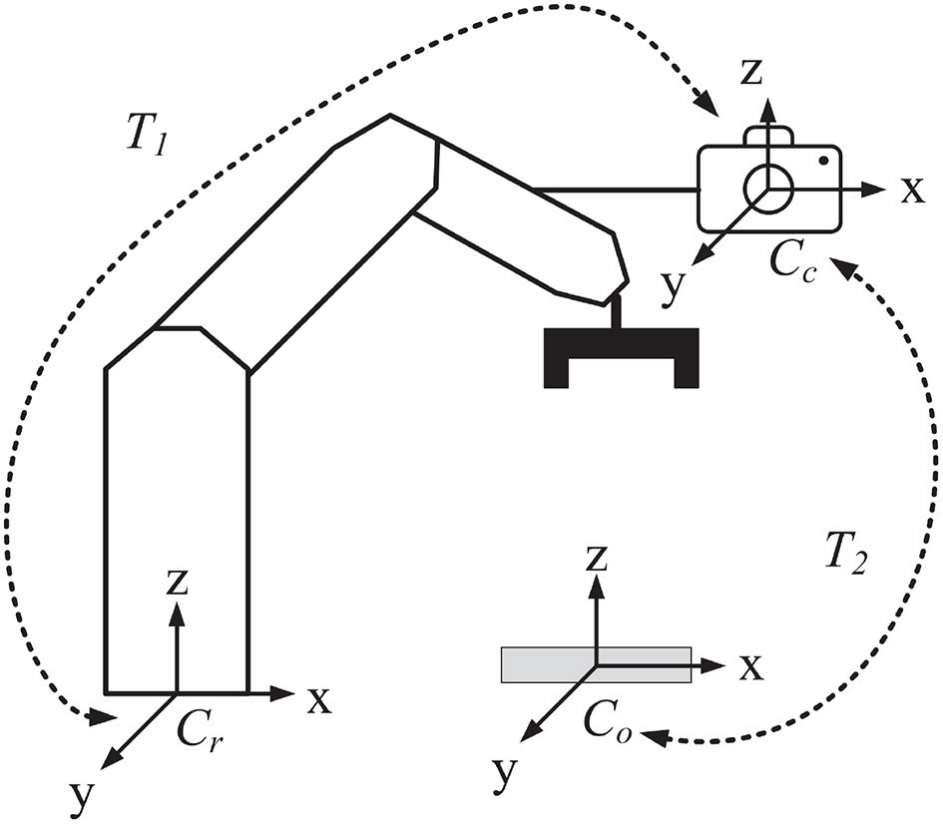
The framework of a vision-guided robotic grasping system.

Object recognition and pose estimation methods can be widely used in robot visual servo systems (Xu et al., 2019, 2020; Wu et al., 2020). In order to explore the feasibility of the proposed method being applied in manufacturing industry scenarios, we have built a vision-guided robotic system for the most common manufacturing task: object grasping. The framework of the system is illustrated in Figure 11. A camera is installed on the manipulator of the robot. Three coordinate systems are labeled including the robot coordinate system *C*_*r*_, the camera coordinate system *C*_*c*_, and the object coordinate system *C*_*o*_. *T*_1_ is the 6D transform from *C*_*r*_ to *C*_*c*_, and *T*_2_ is that from *C*_*o*_ to *C*_*c*_. *T*_1_ is calculated by the famous navy hand-eye calibration algorithm (Park and Martin, 1994) and *T*_2_ is predicted by the proposed algorithm. The object poses relative to the robot are then computed as *T* = *T*_1_ × *T*_2_. The pose matrix is crucial for manipulator path planning and motion control in the grasping task.

**Figure 12:**
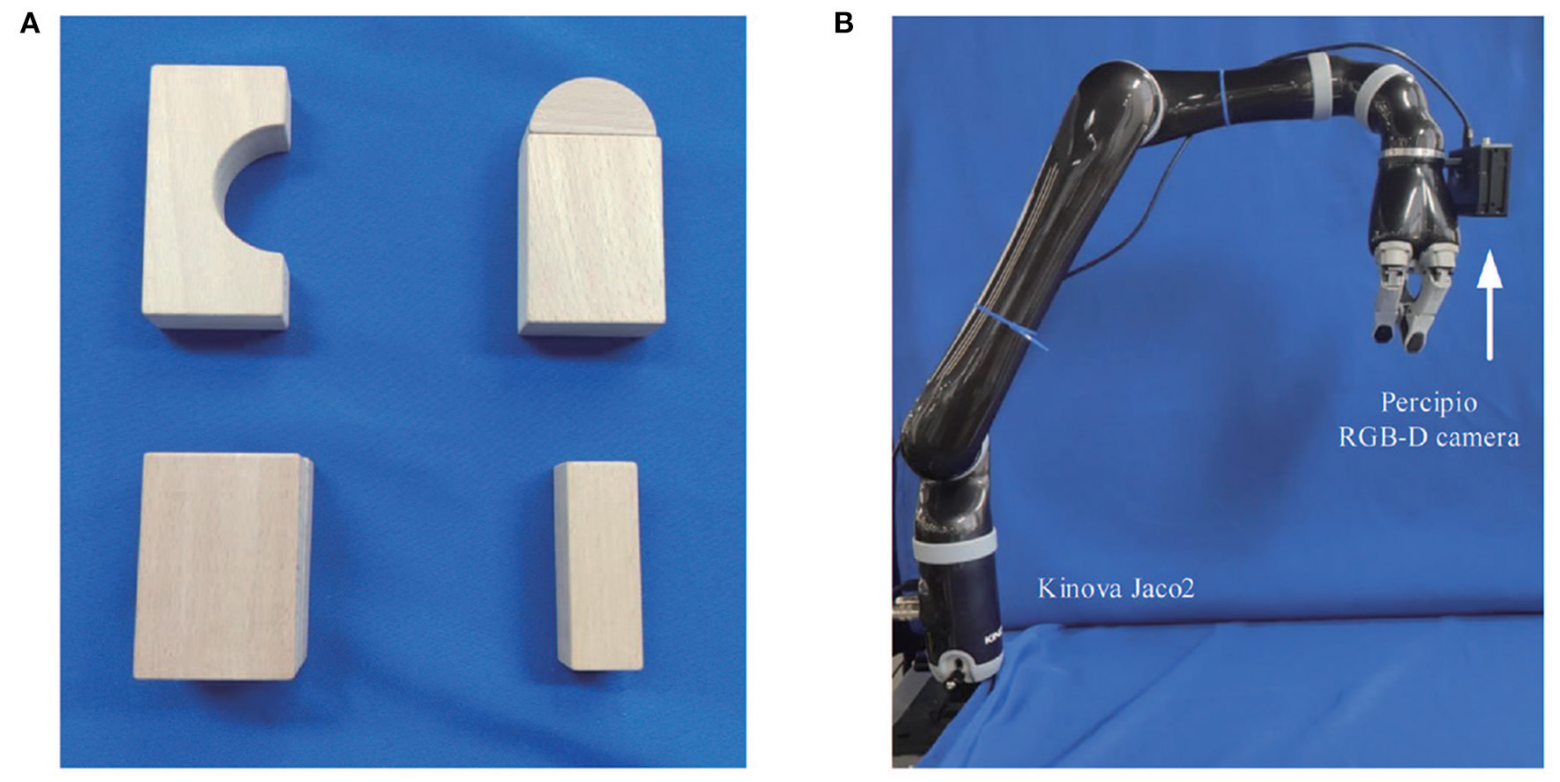
Equipment and target objects used in grasping experiments. **(A)** Some building blocks as target objects, **(B)** The Kinova Jaco2 manipulator with a percipio RGB-D camera installed on the side of the gripper.

The system is composed of a 6 DOF Kinova Jaco2 manipulator and a percipio RGB-D camera FM830-I installed on the side of the gripper, as shown in Figure 12B. The percipio camera utilizes structured light as well as binocular vision to build accurate depth maps. The precision of the captured depth data is up to 1 mm. Morevoer, we also take some building blocks as the target objects in the grasping experiments, as Figure 12A shows.

Before the experiment, first we need to calibrate *T*_1_, then train the semantic segmentation network and 6D pose estimation network. The experiment process is explained as follows: (1) Before grabbing objects, the manipulator should move to a certain position. (2) The RGB-D camera starts to capture images and sends the data to the image processing server. (3) On the server, the RGB-D images are fed into the segmentation network and pose estimation network to predict the 6D pose parameters of the target objects. (4) Based on the predicted transformation matrix, the host computer completes path planning and sends signals to the manipulator making it move to planned positions and performs the operation of grabbing objects, and then placing them in the target area.

**Figure 13:**
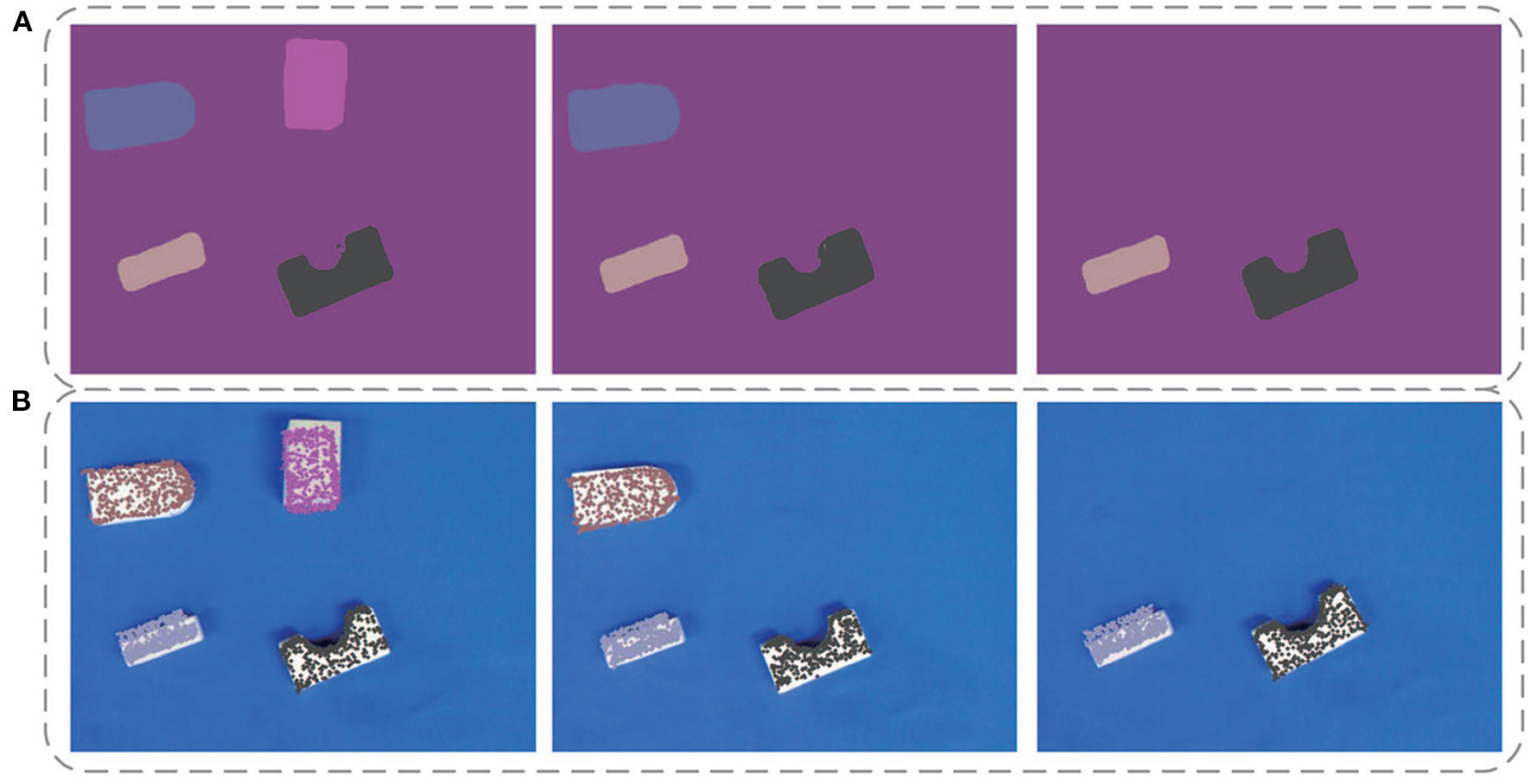
Some experimental results of the robot vision system. Panel **(A)** show the segmentation results, where different colors represent different objects. Panel **(B)** shows the pose estimation results, where the colored points are the 2D projections of the target object point cloud after pose transform.

**Figure 14:**
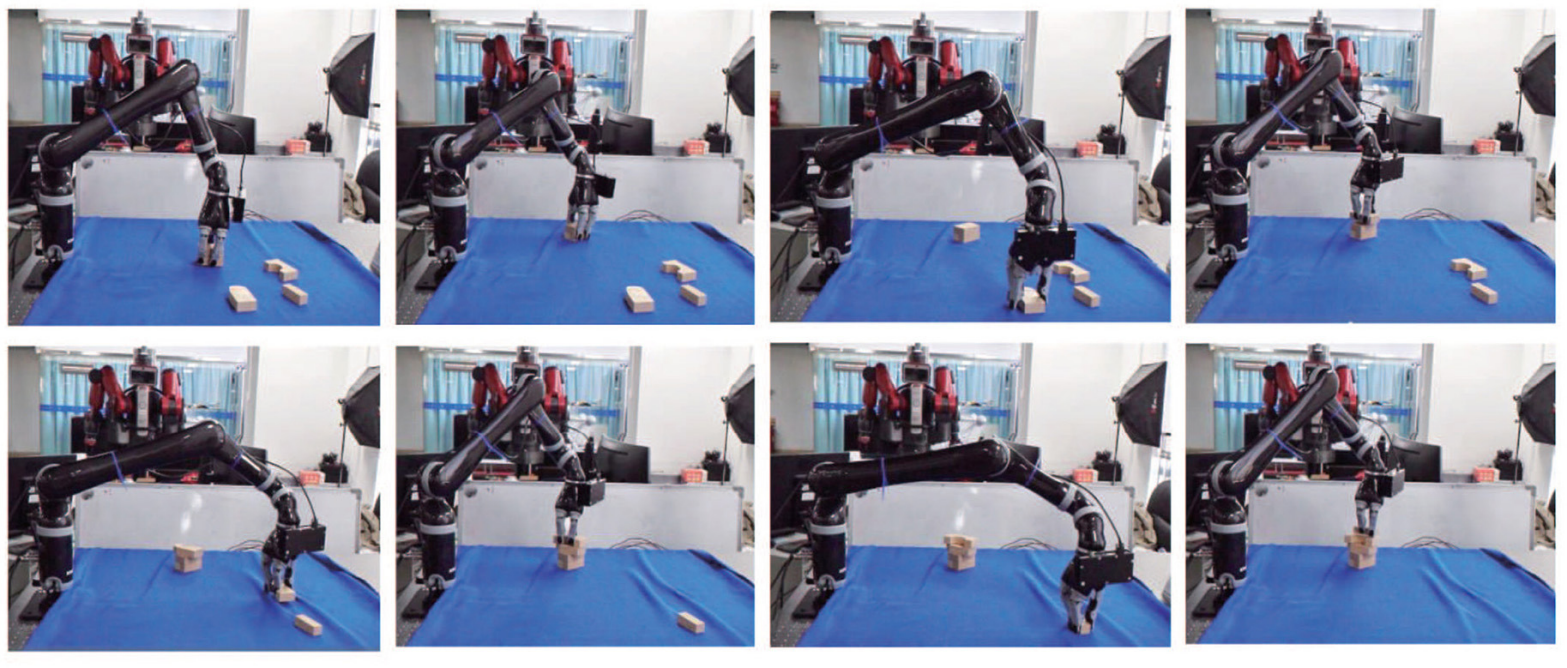
The complete process of picking objects and moving it to target area by the manipulator.

Some experimental results are illustrated in Figure 13. In this case, the segmentation is perfect. However, for some objects, the predictions are not satisfactory. One possible reason is that the poor-textured building blocks may mislead the color feature extractor. In general, the grasping operation runs quickly and smoothly, which, to some extent, verifies the possibility of the new network being applied to all kinds of manufacturing applications. Figure 14 shows the complete process of the grasping experiment.

## 5. Conclusion

This paper presents a new two-stage deep neural network which can efficiently implement object recognition and 6D pose estimation on the input RGB-D images. First, a segmentation network is applied to segment the object from the scene using a densely connected way to fuse different scale features and effectively improve the semantic segmentation results. Second, by introducing the channel and position attention modules, better color and geometric features are extracted for the pose predictor; third, the output pose parameters are further improved by an iterative refinement network. A large number of experiments conducted on two benchmark datasets demonstrated the effectiveness and accuracy of the proposed method in comparison with some state-of-the-art methods. Moreover, a vision-guided robotic grasping system was built, and the grasping experiment has verified the potential of this algorithm being applied in real-time manufacturing applications. Currently, the proposed method still has some problems in dealing with textureless or poor-textured objects. Finer differential geometric features with clear physical meaning and better shape detail are preferred and will be considered in future work.

## Data Availability Statement

The two benchmark datasets LineMod and YCB-Video analyzed for this study can be found at http://campar.in.tum.de/Main/StefanHinterstoisser and https://rse-lab.cs.washington.edu/projects/posecnn/. The building block dataset used in robotic grasping experiments in this paper is built by ourselves and will be made available to any qualified researcher. Further inquiries can be directed to the corresponding author/s.

## Author Contributions

GL conceived the research project. FC, YL, and YF provided support and discussions. GL, FC, and YL proposed the new algorithm. FC, YL, and YF built the whole program and conducted the experiments. GL, FC, and YL wrote the paper. XW and CW performed the English corrections. All authors reviewed and approved the submitted paper.

## Conflict of Interest

The authors declare that the research was conducted in the absence of any commercial or financial relationships that could be construed as a potential conflict of interest.
